# Characterization and Preliminary Biological Evaluation of 3D-Printed Porous Scaffolds for Engineering Bone Tissues

**DOI:** 10.3390/ma11101832

**Published:** 2018-09-26

**Authors:** Chen-Guang Liu, Yu-Ting Zeng, Ranjith Kumar Kankala, Shan-Shan Zhang, Ai-Zheng Chen, Shi-Bin Wang

**Affiliations:** 1 Institute of Biomaterials and Tissue Engineering, Huaqiao University, Xiamen 361021, China; 1611315017@hqu.edu.cn (C.-G.L.); 1400215025@hqu.edu.cn (Y.-T.Z.); ranjithkankala@hqu.edu.cn (R.K.K.); 1611315042@hqu.edu.cn (S.-S.Z.); 2 Fujian Provincial Key Laboratory of Biochemical Technology (Huaqiao University), Xiamen 361021, China

**Keywords:** 3D-printing, poly(lactide-*co*-glycolide), biodegradation, osteoblast growth, tissue engineering

## Abstract

Some basic requirements of bone tissue engineering include cells derived from bone tissues, three-dimensional (3D) scaffold materials, and osteogenic factors. In this framework, the critical architecture of the scaffolds plays a crucial role to support and assist the adhesion of the cells, and the subsequent tissue repairs. However, numerous traditional methods suffer from certain drawbacks, such as multi-step preparation, poor reproducibility, high complexity, difficulty in controlling the porous architectures, the shape of the scaffolds, and the existence of solvent residue, which limits their applicability. In this work, we fabricated innovative poly(lactic-*co*-glycolic acid) (PLGA) porous scaffolds, using 3D-printing technology, to overcome the shortcomings of traditional approaches. In addition, the printing parameters were critically optimized for obtaining scaffolds with normal morphology, appropriate porous architectures, and sufficient mechanical properties, for the accommodation of the bone cells. Various evaluation studies, including the exploration of mechanical properties (compressive strength and yield stress) for different thicknesses, and change of structure (printing angle) and porosity, were performed. Particularly, the degradation rate of the 3D scaffolds, printed in the optimized conditions, in the presence of hydrolytic, as well as enzymatic conditions were investigated. Their assessments were evaluated using the thermal gravimetric analyzer (TGA), differential scanning calorimetry (DSC), and gel permeation chromatography (GPC). These porous scaffolds, with their biocompatibility, biodegradation ability, and mechanical properties, have enabled the embryonic osteoblast precursor cells (MC3T3-E1), to adhere and proliferate in the porous architectures, with increasing time. The generation of highly porous 3D scaffolds, based on 3D printing technology, and their critical evaluation, through various investigations, may undoubtedly provide a reference for further investigations and guide critical optimization of scaffold fabrication, for tissue regeneration.

## 1. Introduction

In recent times, tissue engineering (TE), one of the important concepts of the biomedical field, has garnered enormous attention due to the increase in the demand for organ-replacement treatment, and a shortage of donated organs [1,2,3]. Conceptually, this field involves the replacement/repair of malfunctioned/defect tissues or organs, by artificial biomimetic substitutes, for functional improvement and structural restoration of tissues. This happens to be favorable, by co-culturing appropriate cell phenotypes and scaffolds, in a specific way, through the formation of cell-material hybrid [4,5,6]. Despite the significant advantages and success of the TE field, reconstruction of bone for treating bone defects, with the help of suitable scaffolds that mimic the native bone tissues, has remained a major concern. This is because of the poor biodegradability, inadequate autogenous bone, and presence of immune rejection, in most instances [7,8,9,10]. Thus, the generation of suitable engineered three-dimensional (3D) porous scaffolds, with specific porous architectures that meet the needs of patients with different bone defects, is highly challenging [6,11,12,13].

Several approaches have been applied, so far, in treating bone defects. One of the most-practiced approaches of treatment is the use of metal-based materials, for mounting. These have been widely used as hard tissue replacement materials, especially in the field of plastic surgery and dentistry, because of their high plasticity, excellent mechanical properties, and fatigue resistance [14]. Despite its high mechanical strength and other beneficial attributes, this approach faces certain limitations, such as the forcing of heavy metal rods , during extensive surgical procedures, leading to severe pain that last for several weeks to months, and the rusting of metals in the body, leading to severe sepsis [14,15]. Certain advancements have been made to overcome these limitations, such as the utilization of beta titanium-niobium alloy and other safe approaches. Along this line, Piotrowski and coworkers demonstrated the generation of these materials, and studied the interaction between the implant and the dense bone, by finite element analysis. The stress distribution of the low modulus implant and the reaction of the autogenous teeth have been proved [16]. Further advancements, such as the utilization of traditional ceramic replacement materials, i.e., hydroxyapatite (HA) and tricalcium phosphate (TCP), that are similar to human bone in their composition and structure, have also been made, whose applicability could significantly improve the conductivity of materials and the compatibility of the scaffolds. Therefore, these materials have been widely used as the hard tissue replacement material [17]. In this context, Du and colleagues added HA in glycerol enhanced polyurethane (GCPU) to improve the biomechanical properties of the scaffold and showed that these possessed excellent biocompatibility [18,19]. The scaffold materials implanted in the sprague-dawley (SD) rats, significantly induced new bone production, which proved that the scaffold had shown good repair and regenerative ability. In another study, Hong and coworkers prepared the polyglycolic acid (PGA) and beta-tricalcium phosphate (β-TCP)-based composite scaffolds that had a high porosity, which perforated three-dimensional (3D) porous architectures, by using solvent-casting and particle-leaching approaches [20]. β-TCP had increased the surface roughness of the material and resulted in an excellent therapeutic efficacy, after being implanted in a rat’s femoral defects.

The various traditional approaches for the preparation of bone scaffolds include electrostatic spinning, particle leaching, phase separation, and emulsification/freeze-drying, among others [21,22,23,24,25,26,27]. Despite the success in the formation of porous scaffolds and advances in achieving remarkable progress in TE, some of the unavoidable problems that influence cell growth remain to be addressed. These include issues regarding the compact soft structures, remnants of organic solvent residues, and porogens and rapid degradation which leads to poor cell attachment, deprived cell growth and death, and a poor mechanical strength of the scaffolds, respectively. Often times, these limitations drive the scaffolds towards an irregular growth of bone tissues, due to insufficient time. To this end, 3D-printing technology has emerged as one of the most promising techniques in the late 1980s. It has been used to fabricate geometrically-defined architectures in 3D, for application in various fields, such as pharmaceutical, food, cosmetic, and automobile industries [1,2,3,6,11,12]. From the biomedical point of view, this technology has gained enormous attention in generating 3D-porous architectures. These efficiently improve the physiological relevance of tissues, by overcoming the limitations of various traditional approaches by avoiding the utilization of pore-forming agents (porogens), and organic solvents, in most instances. They also have the ability to achieve control over the 3D architectures, by optimizing the operating parameters and providing high compatibility and reproducibility, among others [3,28,29,30,31].

Herein, the fused deposition modeling (FDM) approach was preferred over other methods of 3D printing, to prepare bone tissue substitute materials, as it has a more simplified operation process. It provides a greater convenience and flexibility in the processing of the scaffold, provides excellent reproducibility (with the help of computer-aided design (CAD) processing), and it is eco-friendly, i.e., does not use an organic solvent [32]. On the other hand, PLGA, a United States Food and Drug Administration (US FDA)-approved polymer, was used as a raw material as it has better mechanical properties, is non-toxic, has a good biocompatibility, and is conducive to the promotion and induction of tissue repair [33,34].

Motivated by these facts, we demonstrated the fabrication of porous scaffolds, using 3D-printing technology, for engineering bone tissues. Initially, the prepared scaffolds were systematically characterized using various physical characterization techniques. Further, the biodegradation of the scaffolds, in simulated body fluids (hydrolytic as well as enzymatic degradation) was critically evaluated. This was done to demonstrate the morphology, as well as the physicochemical properties of the scaffolds, and evaluate the fabrication, mechanical properties, degradation rates, toxicity, and biocompatibility of the PLGA scaffolds. Prior to the bioefficacy evaluation of bone tissue growth, the cytocompatibility of these scaffolds was evaluated in a cell model, i.e., mouse fibroblast cells (L929 cell line) (normal fibroblasts), using an optical microscope. This cell line was used as it is the most preferred model for demonstrating the compatibility of materials. On the other hand, the embryonic osteoblast precursor cells (MC3T3-E1) were used to evaluate the bone growth, as these preosteoblasts have been extensively used as an in vitro model, revealing their suitability for testing the efficacy of biomaterials. Here, the cell adhesion rate, proliferation, as well as a distribution study of MC3T3-E1 cells on the scaffolds, were shown, demonstrating the underlying implications of the porous scaffolds on engineering bone tissues [35].

## 2. Materials and Methods

### 2.1. Materials

PLGA copolymer (75:25, Mol. Wt. = 60 kDa) was purchased from the Daigang Biological Co. Ltd. (Jinan, China). Potassium bromide (KBr), absolute ethanol (99.8% purity), tetrahydrofuran (THF), and dichloromethane (DCM, 99.8% purity) were purchased from the Sinopharm Chemical Reagent Co., Ltd. (Shanghai, China). Lysozyme ~7000 U/mg was purchased from Sigma-Aldrich (St. Louis, MO, USA). All other chemicals, solvents, and reagents were of analytical purity and used without any further purification. Cell lysate, Roswell Park Memorial Institute 1640 (RPMI-1640), α- minimum essential medium (α-MEM), albumin bovine V, and Fetal bovine serum (FBS) were acquired from Gibco (USA). Cell Counting Kit-8 (CCK-8) was purchased from KeyGEN BioTECH, Jiangsu, China. Bradford Protein Quantification Kit was purchased from YEASEN, Shanghai, China.

### 2.2. Preparation of PLGA Scaffolds

A commercial 3D printer (Regenovo 3D Bio-Architect, Hangzhou, China) was used to print the 3D-porous scaffolds based on the FDM process. Herein, PLGA was added into the high-temperature cylinder (3D Bio-Architect), the conditions provided were initially optimized, and the printing process was continued. Moreover, the specifications relevant to the gel dispenser were adjusted such that the width, length, and height of the porous scaffold were 10 mm × 10 mm × 4 mm. Prior to printing the scaffolds for the growth of bone tissues, a set of operating parameters, such as temperature, pressure, velocity, thickness, and filling distance, corresponding to the inner diameter of the nozzle, were examined and critically optimized (Table 1).

It is evident from the literature that the main factors affecting the scaffold formation and its properties are the thickness of the scaffold, the structure, and the anisotropy of the material. Therefore, the performance of the scaffolds on the above-mentioned aspects, was investigated. For the other conditions, the scaffolds with different thickness (0.2, 0.3, and 0.4 µm), printing angle (0°/90°, 0°/60°/120°, and 0°/45°/90°/135°) and porosities (45%, 52%, 59%, 66%, and 73%) were prepared, respectively. Furthermore, the porosity of the scaffolds was measured by the density method, through a conversion of the density of the PLGA raw material (ρ = 1.3 g/cm^3^). The density of the scaffolds and their mechanical properties were also tested.

ρ_s =_ 4 *m*/π*d*^2^*h* (1)

Ф(%) = (1 − ρ_s_/ρ) × 100 (2)

where ρ_s_, *m*, *d*, *h*, and Ф is the density, mass, diameter, height, and porosity of the scaffolds, respectively.

### 2.3. Physical Characterizations

Samples were prepared according to the requirements of the ISO 604: 2002 standard and the mechanical properties of the porous scaffolds were tested by a universal testing machine. Initially the force attenuation rate was set to be 60%, and the sample was compressed with a speed of 1 mm/min. The results of compression strength, and yield stress were recorded. The stress-strain curve was obtained by inputting the actual area, as well as the height of the scaffolds, and manually adjusting the height of the pressure bed. When the obtained results were in agreement with the ISO 604: 2002 standard guidelines, the data were exported. The effects of various printing conditions, i.e., the layer thickness of 0.2, 0.3, and 0.4 mm, printing angle of 0°/90°, 0°/60°/120°, and 0°/45°/90°/135°, and porosity of 45%, 52%, 59%, 66%, and 73% on the mechanical properties, were also determined. The functional groups of the scaffolds and the raw materials were tested by the attenuated total reflectance Fourier transform infrared (ATR-FTIR, Thermo Scientific NICOLET iS 50, Waltham, MA, USA) spectroscopy, to detect changes in the functional groups of the samples, before and after preparation, at a specified wavelength range of 4000–400 cm^−1^. X-ray diffraction (XRD, Rigaku Corporation, Tokyo, Japan) data were recorded on an XRD spectrometer, equipped with graphite monochromatized copper-Kα radiation (λ = 0.15405 nm, 2-theta ranging from 10° to 35°). The degradation behavior and thermal stability were determined by differential scanning calorimetry (DSC) technique. The samples (~10 mg) of raw PLGA, as well as PLGA 3D scaffold, were placed in the alumina crucible and subjected to heat, on a scale of 20 to 200 °C, at a heating rate of 10 °C/min, under nitrogen as a carrier gas (20 mL/min).

### 2.4. Degradation Analysis

The degradation of the scaffolds was tested by immersing them in the phosphate-buffered saline (PBS, pH-7.4, hydrolytic) and enzyme dispersed PBS solutions (enzymatic), at a ratio of 1:10 (V:M), and placing them in an incubator, maintained at a constant temperature (37 °C) and oscillation rate (60 r/min). The study was continued by replacing the equal amounts of the respective buffer solutions, at predetermined time intervals. Further, the samples were flushed with deionized water, and the weight loss rate, change of pH value of the buffer, mechanical properties, surface morphology, glass transition temperature (*T*_g_), molecular weight and its distribution, and thermal decomposition temperature were detected, at various predetermined time intervals, i.e., at every alternative week from the starting period (0th, 2nd, 4th, 6th, 8th, and 10th week). The surface morphology of the scaffolds was observed under the microscope, after being washed and dried with deionized water. 

The weight of each scaffold, before and after the degradation experiments, was determined accurately and recorded as *W*_0_ and *W*_t_, respectively. The weight loss was calculated by the following formula: *L*%= (*W*_0_ − *W*_t_) /*W*_0_ × 100.

The mechanical properties of the porous scaffolds were tested by a universal testing machine at 0th, 2nd, 4th, 6th, 8th, and 10th week, respectively (detailed testing procedure has been discussed in Section 2.3). The experimental process included washing the scaffold thrice with deionized water, followed by measurements of the area and thickness of the scaffold, after freeze-drying. The scaffolds were then placed on the test bed, and the scaffold material was compressed at the speed of 1 mm/min, and the corresponding stress-strain curve was finally determined.

In the 2nd, 4th, 6th, 8th, and 10th week of the degradation experiment, the samples (10 mg) were placed in the alumina crucible, and subjected to the experimental temperature range, from 0 to 200 °C, at the heating rate of 10 °C/min, under nitrogen atmosphere, at a flow of 20 mL/min, for the DSC analysis. Similarly, the differential thermal analysis /thermogravimetry (Shimadzu(China), Shanghai, China) (DTA/TG) was performed at the scanning range of 40–500 °C, at a heating rate of 10 °C/min, under nitrogen flow at the rate of 20 mL/min.

The molecular weight of the PLGA and its distribution of the scaffolds were measured by gel permeation chromatography (GPC). The freeze-dried PLGA scaffold was washed with deionized water and dissolved in THF at a proportion of 5% (m/V). The flow rate was 1 mL/min, and the temperature was maintained at 40 °C. The weight-average molecular weight of PLGA was calculated with polystyrene (PS), as the standard sample.

### 2.5. Bioefficacy Measurements

#### 2.5.1. Cytotoxicity Test

*Preparation of the extract of the scaffold:* Initially, the prepared scaffolds were soaked in 75% ethanol, for 2 h. After drying, the scaffolds were UV–irradiated, for 2 h on each side, to sterilize the materials further. The scaffolds were soaked in RPMI-1640 medium, at 37 °C, 60 r/min, for 72 h, and the scaffold-free media were filtered through a 0.22 µm filter membrane, to prepare a scaffold leaching solution, at a concentration of 0.1 g/mL.

*Cell inoculation:* L929 cell line was inoculated at a density of 2 × 10^5^ cells/mL, into a 96-well culture plate and then incubated, for 24 h, for proper cell attachment. Then, the media was removed, and the cells were washed twice with PBS. Various concentrations of the scaffold leaching solution (10, 50, and 100 mg/mL) were added as the experimental groups, along with the fresh medium, and phenol 0.64% medium, as a negative control group and the positive control treatment group, respectively. After 24, 48, and 72 h of exposure, the media in the wells were removed, and 10% of CCK-8 working solution (100 µL) was then added to each well. Further, the cells were incubated in the dark, for 4 h. Finally, the absorbance, at a wavelength of 450 nm, and the relative proliferation rate (relative growth rate, RGR) was calculated by using the following formula.

RGR% = absorbance (OD_450_)/control group absorbance value (OD_450_) × 100 (3)

#### 2.5.2. Protein Adsorption Kinetics

Here, FBS was used as a model for the protein adsorption experiments. Initially, the printed scaffolds were placed in a 24-well plate and added with 1 mL of α-MEM media, containing 10% FBS. They were then incubated in a shaker, maintained at 37 °C (60 rpm) for 0.25, 0.5, 1, 2, 3, 4, and 5 h, respectively. Further, the scaffolds were removed and washed once with PBS and then transferred to a new plate and added with 1 mL of protein eluting buffer (urea 8 M, Tris 0.1 M, pH-8.6), for 20 min, at room temperature. Finally, the protein content was detected with the G250 staining solution (Bradford) by correlating the absorbance with the standard curve of albumin from bovine serum (BSA).

In addition to the protein adsorption, we also evaluated the secretion of cell proteins that are responsible for the generation of extracellular matrix (ECM), in the scaffolds. After the scaffold was pretreated by 75% ethanol, for 2 h, and then sterilized by irradiating with UV irradiation, for 2 h on each side, it was placed in a 24-well plate. The cell suspension at a density of 6 × 10^5^/mL was added and then incubated for 24 h. The media was discarded, and the fresh medium containing 5% FBS was then added and incubated further for 12 h. Later, the media was replaced with the serum-free media and further incubated for 24 h. Sample aliquots (20 µL) were then collected and added with 200 µL of Bradford reagent. Then, the absorbance was detected, and the protein concentration in the sample, was calibrated with the plotted standard curve. Eluting scaffolds with protein eluent (urea 8 M, Tris 0.1 M, two disulfide 0.01 M) and Bradford reagent were used to detect the concentration of the protein contained in it. The total amount of protein secreted by the cells represents the amount of protein contained in the supernatant, as well as the protein content on the scaffold.

#### 2.5.3. Cell Adhesion Rate

The adhesion rate of the cells, to the scaffolds, was demonstrated as follows. Initially, the scaffold was activated by immersing in a 75% ethanol solution, for 2 h, and then sterilized by irradiating with UV irradiation, for 2 h on each side. The scaffolds were then fixed to the bottom of the 24-well plate and soaked with 1 mL of the α-MEM medium, for 24 h. The MC3T3-E1 cells, at a density of 3 × 10^5^ /mL, were seeded onto the scaffolds, along with the blank group, and were incubated. The scaffolds were separated and washed with the PBS, at predetermined time intervals of 0.5, 1, and 2 h, and the unattached cells were collected and counted. The adhesion rate of the cells was calculated by the following formula.

Cell adhesion rate (%) = (quantity of inoculated cells − non adherent cells)/cell inoculation volume × 100(4)

#### 2.5.4. Cell Proliferation Study

Furthermore, the proliferation of cells over the scaffolds was measured by following the procedure below. The activated, as well as the sterilized scaffolds, were fixed at the bottom of a 24-well plate. Then, the MC3T3-E1 cells were seeded at a density of 2 × 10^6^/mL, in each well. The treatment, without the scaffold, was used as the blank control group. Later, the scaffolds were removed and transferred into the fresh culture plate. After 1, 4, and 7 days of exposure, the media was then removed, and the scaffolds were washed twice with PBS. Then, 10% CCK-8 working solution was added, and the mixture was incubated in the dark, for 4 h. Finally, the absorbance was recorded at a wavelength of 450 nm.

#### 2.5.5. Cell Distribution Study

MC3T3-E1 cells were seeded at a density of 5 × 10^5^/mL, in each well of a 24-well plate, which was fixed with the scaffolds. Further, the mixture of cells and the scaffolds were incubated (37 °C and 5% CO_2_) for 24 h, for proper cell attachment. After culturing for 3 and 6 days, respectively, the media was removed, and the scaffolds were washed with PBS. Then, the cells were fixed and 0.01% acridine orange dye was added, for 1–2 min. After 3 washes with PBS, the scaffolds were then observed under confocal laser scanning microscopy (CLSM, TCS SP5, Leica, Wetzlar, Germany), at an excitation wavelength of 488 nm, and an emission wavelength of 633 nm.

### 2.6. Statistics Analysis

All results were subjected to statistical significance tests, and are presented as the mean *±* standard deviation (SD) in the Results and Discussion section. The statistical analysis of all the experimental data was performed using statistical product and service solutions (SPSS) version 19.0. Analysis of variance (ANOVA) single-factor analysis was conducted at a defined level of statistical significance of *p* < 0.05. 

## 3. Results and Discussion

### 3.1. Physical Characterizations

**Figure 1:**
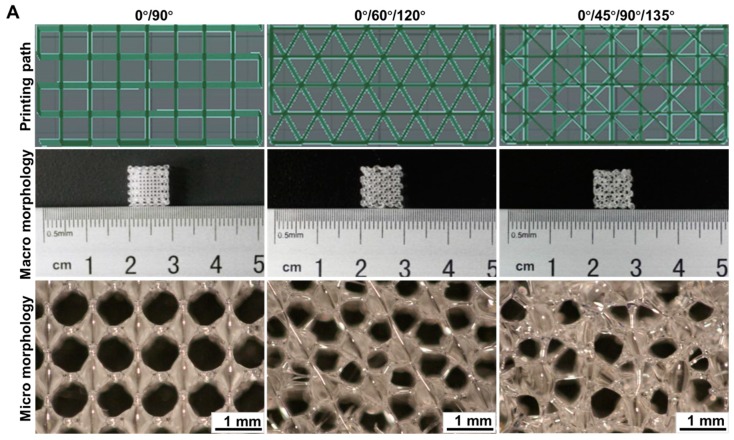

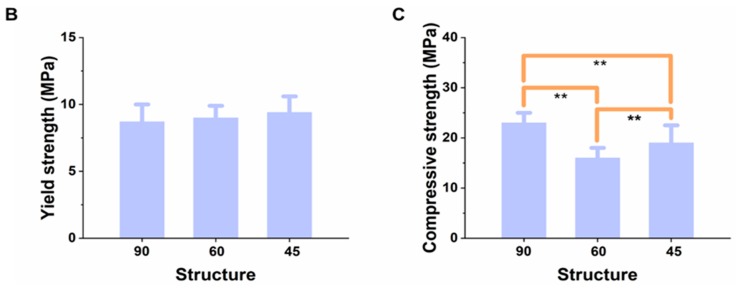
(**A**) Printing path, macro—as well as micro—morphology of the PLGA scaffolds. Change of (**B**) yield stress, and (**C**) compressive strength, at various printing angles. The size of the scaffolds was 10 mm × 10 mm × 4 mm, with an area of error of about ±0.05 cm^2^, (*n* = 5). ** represents *p <* 0.01.

Indeed, the cells could significantly respond to the signals in the scaffold’s ECM environment, and could convert them into the intracellular signals that affect cell functions, including their adhesion, proliferation, differentiation, and migration. The appropriate changes in the topological structure of the biomimetic material were considered to be an effective means of regulation for engineering tissues [17,36]. Therefore, three different types of angle variations were chosen for printing the 3D structures—0°/90°, 0°/60°/120°, and 0°/45°/90°/135°, were used as the objects of investigation. Under the same conditions, the compressive strength and yield stress of these structures were tested. As shown in Figure 1A, it was evident from the microstructure of the scaffolds that their 3D architectures, printed at a different angle, were relatively regular and had a uniform final size with a clear boundary, demonstrating the feasibility of the FDM technique. Moreover, the deposition path of the scaffold was consistent with the path designed by the CAD, which resulted in the uniform-sized pores. Compared to the structure of the 0°/60°/120°and that of 0°/45°/90°/135°, the morphology (with respect to thickness), as well as the porous arrangement, was more regular, with a better reproducibility, in the case of the 0°/90°printing angle. The experimental results of the scaffolds, with the structure of 0°/90°, 0°/60°/120°, and 0°/45°/90°/135°showed that the obtained yield stress values were 8.70 *±* 1.28, 8.93 *±* 1.01, and 8.76 *±* 0.86 MPa (Figure 1B), and the compressive strengths were 23.58 *±* 1.36, 18.91 *±* 2.03, and 15.49 *±* 1.01 MPa (Figure 1C) respectively. These results were in agreement with the data reported by Zein and coworkers [37]. In another study, Shkarina and colleagues fabricated the polycaprolactone (PCL) scaffolds by an electrospinning method, in which a fibrous scaffold (wPCL) with parallel fiber alignment in one direction, showed a significant tensile strength [38]. In addition, it was evident from Table 2, that the pore size and the column diameter of the three structures were basically in the same range (the thickness and porosity of the scaffolds were 0.2 mm and about 67%, respectively, *n* = 3). Therefore, the variation in topological structures was the basis for the difference in the mechanical properties of the scaffolds. Moreover, the pore size of the scaffold at 300–700 µm was more conducive to cell adhesion, proliferation, and matrix synthesis. So, this performance of scaffolds was achieved only when the porosity was about 67% [39].

**Figure 2:**
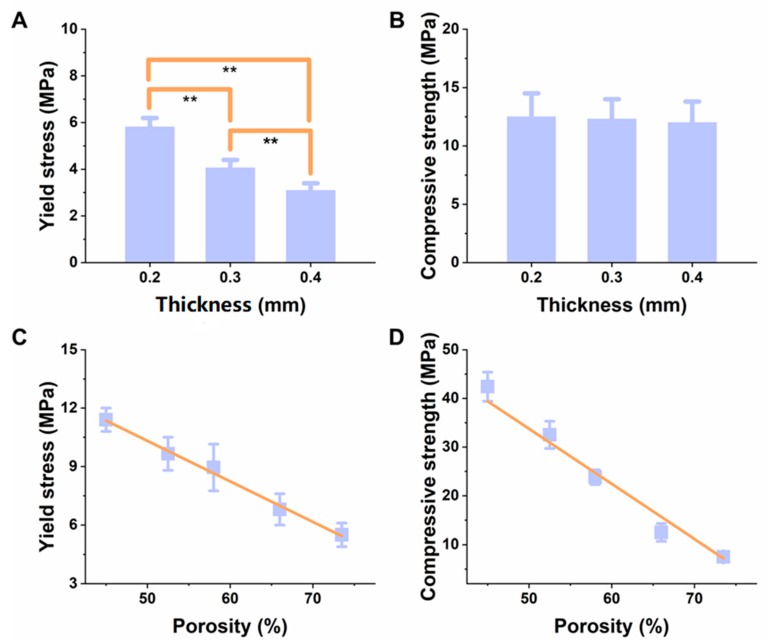
Change of (**A**) yield stress, and (**B**) compressive strength at a varied thickness (0.2, 0.3, and 0.4 mm) and porosity (45%, 52%, 59%, 66%, and 73%), (*n* = 5). Change of (**C**) yield stress, and (**D**) compressive strength at a varied porosity (45%, 52%, 59%, 66%, and 73%),** represents *p <* 0.01.

More often, the difference in thickness during the printing of the scaffolds would affect the interlayer space and the horizontal spacing, so that the scaffolds result in different pore sizes and altered mechanical properties. In order to determine the appropriate thickness, we selected three different thickness (0.2, 0.3, and 0.4 mm), and the respective mechanical properties (i.e., the yield stress, as well as compression strength) of the scaffolds were determined, under the same conditions [37,40]. Accordingly, the porosity of the scaffolds at different thickness was at around 67%. The experimental results illustrated that the compressive strength of the three groups had shown no noticeable difference, with an increase in thickness, i.e., 12.69 *±* 1.59, 12.50 *±* 1.28, and 11.99 *±* 1.30 MPa, respectively (Figure 2B). However, a significant decrease in the yield stress of the scaffolds was gradually observed, with a change in the thickness of the scaffolds (5.78 *±* 0.60, 4.12 *±* 0.58, and 3.15 *±* 0.43 MPa, respectively (Figure 2A)), revealing that the scaffold had shown improved mechanical properties, at a thickness of 0.2 mm, which were appropriate for the mechanical strength of the cancellous bone [41]. The continuous increase of the thickness was not conducive to the degradation of the scaffold, and to the adhesion and proliferation of the cells [42]. Therefore, a thickness of 0.2 mm was selected as the optimal thickness for the experiments.

In general, the gradual increase of porosity, results in a rapid degradation of the scaffolds, and poorer mechanical properties of the scaffolds. The results of Figure 2C,D show that with an increase of porosity, the compressive strength and yield stress tended to decrease gradually, which is in accordance with the results of Lam and coworkers [43]. When the porosity was about 66%, the mechanical properties, i.e., compressive strength, as well as the yield stress values, were 12.69 *±* 1.59 MPa, and 5.78 *±* 0.60 MPa (*n* = 5), respectively. Accordingly, the compression strength of the human cancellous bone should have been around 4–12 MPa, under normal conditions. Therefore, the compressive strength and the yield stress of the scaffold materials were in the acceptable range of that required for the mechanical stability of human cancellous bone [44].

Through the optimization of the various properties, such as thickness, structure, and porosity, the scaffolds were prepared for good comprehensive performance. The optimized specific parameters included a porosity of 66%, a thickness of 0.2 mm, and a printing angle of 0°/90°, possessing a compressive strength and yield stress of 12.69 and 5.78 MPa, respectively. Under these conditions, the mechanical strength of the scaffold had reached the requirement of the cancellous bone, by maintaining the external pressure as a scaffold material. The scaffolds showed higher mechanical properties, compared to that of the scaffolds made of starch-based polymers (cornstarch, dextran, gelatin) and PLGA composite scaffolds, prepared by a low-temperature deposition technique [32,43]. Another interesting feature of this approach was the convenient method for fabrication of the required designs, with easy implementation of variations in the physicochemical, as well as mechanical properties for practical applicability, by a simple alteration of the printing conditions.

**Figure 3:**
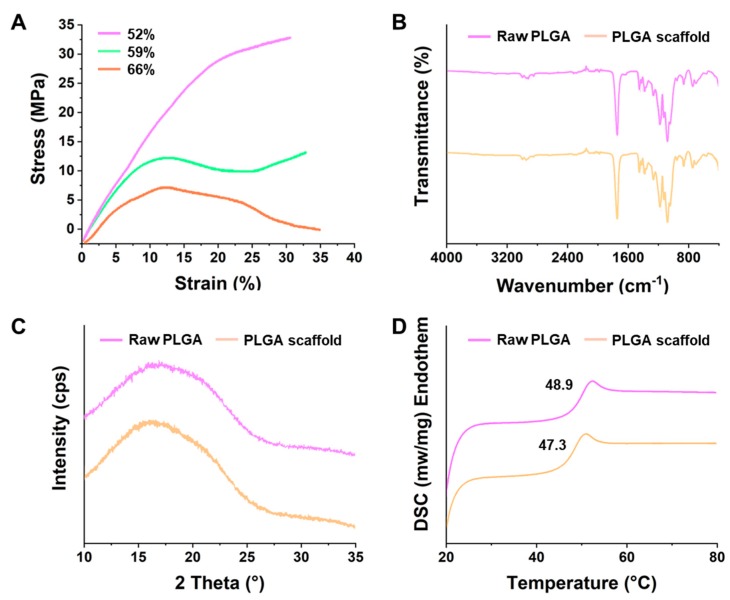
(**A**) Strain-stress curves at different porosity levels of the scaffolds. (**B**) FTIR spectra, (**C**) XRD curves, and (**D**) the DSC curves of the raw PLGA and the PLGA scaffold.

It is evident from Figure 3A that the porosity of the scaffolds had shown significant influence on the yield stress of the scaffold. At less than 60% of porosity (52%), it had shown no yield phenomenon. When the porosity increased from 59% to 66%, the yield phenomenon gradually appeared, and was more obvious at 66% of porosity. The obtained results were different from the data obtained from Zhang and coworkers [45]. The reasons might be due to the utilization of a different approach for the scaffold preparation and difference in the range of porosity (60%–72% and 78.8%–94.5%) [45]. In addition, from the standard stress-strain curves, Young’s modulus of the scaffolds, at a porosity of 52%, 59%, and 66%, was 120.80, 12.48, and 1.50 MPa, respectively, indicating that Young’s modulus of the scaffold was significantly reduced with an increase in porosity [46,47]. 

Furthermore, the scaffolds prepared at the optimized printing conditions were systematically characterized using various physical characterization techniques. This was done to illustrate any changes in the PLGA, during fabrication, by comparing the respective results of these scaffolds with the raw PLGA. Figure 3B compares the infrared spectral vibration of the molecules of raw PLGA and its respective scaffold, prepared by an FDM-based 3D printing method. The characteristic peak of PLGA at around 1428 cm^−1^ is ascribed to the stretching vibration of the –O–CH_2_–, which was a unique structure of glycolic acid (GA). The peak at 1765–1645 cm^−1^, corresponds to the C=O stretching vibration. Moreover, several asymmetric peaks, at around 1330~1050 cm^−1^, were attributed to the –C–O–C–, in consideration of the existence of C=O [48]. It was evident from the (ATR-FTIR) spectra that no significant difference in the absorption peaks was observed, demonstrating that the high-temperature melting during the 3D modeling had no effect on the chemical structure of the PLGA. Moreover, it clarified that the process of the molten-deposition preparation approach might not have resulted in any changes of the properties and caused any damage to the materials. Figure 3C shows no obvious change in the XRD spectra of the scaffold material, as compared to that of the raw material, in which, both show a halo-shaped characteristic peak, indicating that the PLGA remained consistent in the amorphous state, before and after the preparation [35]. Furthermore, the thermal behavior, as well as the thermal phase transition analysis of the copolymers and the scaffolds, were determined by a DSC analysis (Figure 3D). This is often determined by the glass transition temperature (*T*_g_) of the polymer, which reflects the rearrangement of the polymeric segments, where the atom or group of atom can only vibrate at its equilibrium position. Compared to the raw PLGA, the 3D-printed PLGA scaffolds had shown only a small change in the *T*_g_ of the PLGA, after the printing was done, which revealed the changes in the structural rearrangement (Figure 3D). This data is in accordance with the results of Zein and colleagues [37], illustrating that the high-temperature treatment process had no obvious effect on the properties of the PLGA, but it might have resulted in only certain physical changes of the structural rearrangement.

### 3.2. In Vitro Degradation of the PLGA Scaffolds

**Figure 4:**
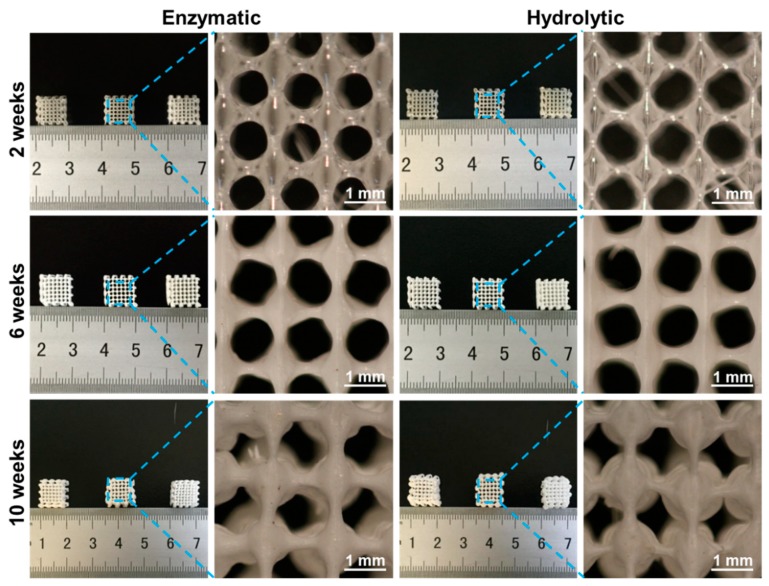
Macro-and-Micro-morphological view of the PLGA scaffolds, after enzymatic and hydrolytic degradation, at predetermined time intervals (2, 6, and 10 weeks of exposure time). The size of the scaffolds before degradation was 10 mm × 10 mm × 4 mm, with an area error of ±0.05 cm^2^, (*n* = 5).

Biodegradation is the most crucial property to be considered while preparing biomedical devices. Moreover, the initial understanding of the degradation process of TE scaffolds is critical, as it plays a significant role in utilizing them and determining their tissue regeneration efficacy [49]. It should be noted that, more often, the ideal scaffolds used for engineering tissue should possess controllable degradation, whose degradability rate should be rehabilitated to the speed of the tissue regeneration process [50]. In this study, scaffolds fabricated at few optimized parameters, were subjected to degradation studies in various fluids, such as normal buffers (hydrolytic) and enzyme-dispersed buffers (enzymatic) mimicking the physiological fluids. Furthermore, various physicochemical attributes were also measured, such as changes in weight, mechanical properties of the scaffolds, and the pH of the medium, correlating the effect of the medium on the scaffolds. It is evident from Figure 4 that the scaffolds had resulted in a change of color, i.e., becoming white from the original transparency, which revealed the signs of degradation of the scaffold. In addition, the regular size of the pores was also disordered, resulting in an increase of the scaffold volume. These consequences might be due to the interaction of the scaffolds with the water molecules, and gradual decomposition of the material, into small molecules. At the early stages of degradation (at 2nd week of incubation period), the morphology of the scaffold remained regular and complete. However, a slight disintegration began in the next four weeks (at 6th week of incubation period). Moreover, the texture of the scaffold began to become more fragile, and the scaffold began to deform from 10th week, in both enzymatic as well as the hydrolytic degradation media.

**Figure 5:**
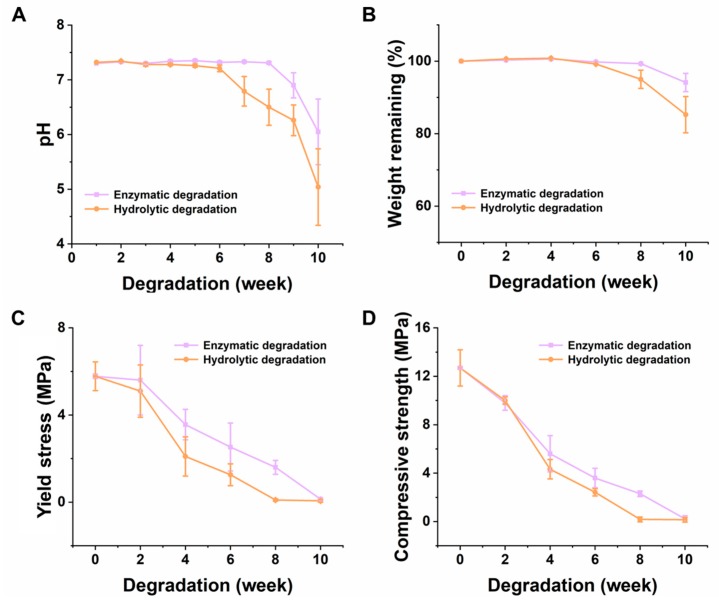
The effect of various physicochemical properties during the degradation of the PLGA scaffolds. Change of (**A**) pH value, (**B**) weight, (**C**) yield stress, and (**D**) compressive strength, with time (*n* = 5).

As shown in Figure 5A, the pH value of the buffer solution remained constant at the initial stage of degradation, indicating that the degradation rate of the scaffold corresponding to the incubation time period is slow. Further, the pH began to decline from the sixth week due to the generation of lactic acid (LA) from the PLGA, revealing the degradation of the scaffold, due to the production of carbon dioxide and water, after the degradation of the scaffold. Moreover, the decline in pH in the hydrolysis medium (pH-5.04) was faster than that of the enzymatic (pH-6.05), after a ten-week degradation period because the PLGA is a non-enzymatic degradable polymer. Therefore, the existence of the enzyme in the degradation medium might not necessarily have promoted the fracture of the ester bond, but could only accelerate the dissolution of a low-molecular-weight degradation of the products of PLGA. Therefore, the autocatalytic effect caused by the decrease of the pH in the scaffold, had slowed down, and the hydrolysis had also slowed down, on the whole [51]. Although the change of pH in the later stage of degradation was noticeable, the acidic substituents produced by the scaffold degradation could be balanced by their penetration into the surrounding environment, thus, effectively alleviating the autocatalytic effect and the subsequent degradation rate of the scaffolds [52]. According to Figure 5B, it is evident that there was no sign of changes in the quality of the scaffolds, in the early stage, till the sixth week of the degradation study, in both enzymatic as well as hydrolytic mediums. However, with the decrease of pH, the quality of the scaffold began to decline, indicating that the degradation rate of the PLGA, in the degradation process, was first slow and then augmented, i.e., there was an accelerated degradation of the material [53]. In the 10th-week of the study, the mass loss rates of the two degradation methods were relatively small, and the quality of the scaffolds, in the enzymatic and hydrolytic studies, decreased from 100% to 94.12% and 85.24%, respectively.

With the degradation of the scaffold in any medium, the mechanical properties of the scaffold gradually become poorer. Thus, this trend of the mechanical properties of the scaffolds could also be used to correlate the degradation rate of the scaffolds. Figure 5C,D shows the decreasing levels of the yield stress and the compressive strength of the scaffold, gradually, with time. The overall degradation rate was consistent, and the degradation trend in the hydrolysate and in the enzymatic, was approximately the same. However, the rate of degradation in the hydrolysate was faster than that in the enzymatic study, after 4 weeks. The compressive strength decreased from 12.69 MPa to 0.22 MPa and 0.15 MPa, in the enzymatic and hydrolytic media, respectively. A similar trend was evidenced in the case of the yield stress of the scaffolds, which decreased from 5.78 MPa to 0.14 MPa and 0.06 MPa, in the enzymatic and hydrolytic media, respectively. This might be due to the change of the molecular weight caused by the hydrolysis of the ester bond, in the degrading solution. This could have directly led to the worsening of the mechanical properties, due to the gradual change in the texture of the scaffolds and the weakening of the intermolecular forces [54,55]. However, it should be noted that when the scaffold is implanted in the body, as it would get degraded, new tissue would gradually replace the empty space in the scaffold, and the space produced by the degradation, and strengthen the supporting effect. Generally, a fibrous matrix is formed in the first three weeks, which effectively alleviates the damage caused by the decrease of mechanical properties, in the later period. Therefore, the rate of degradation of prepared scaffolds is suitable for providing support in the first three or four weeks, leading to no harmful effects during the formation of new tissue [56].

**Figure 6:**
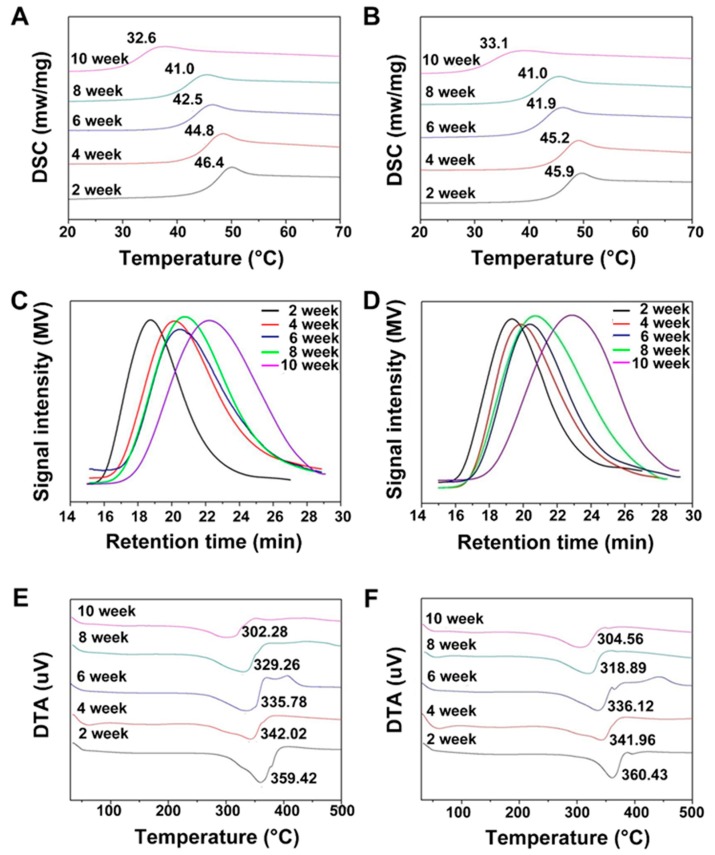
(**A**,**B**) DSC curves, (**C**,**D**) molecular weight distribution, and (**E**,**F**) DTA curves of the enzymatic (**A**,**C**,**E**) as well as the hydrolytic (**B**,**D**,**F**) degradation studies.

Furthermore, various physical characterization techniques were used to systematically demonstrate the fate of the physical properties of the scaffolds, after the enzymatic and the hydrolytic degradation procedures. Figure 6A,B illustrate that the *T*_g_ of the scaffold gradually decreased with increase in time of exposure to the degradation medium, indicating that the free melting-temperature of the polymer chain segments gradually decreased. That is, the temperature conditions required for the transition from the glass state to the high elastic state, were reduced. In addition, there was always only one exothermic peak in the DSC curve, indicating that the scaffold material was always amorphous, during degradation.

PLGA is generally hydrolyzed, itself, by means of an ester bond cleavage. As the fracture site of the ester bond was not explicitly fixed, the result might have been irregular. At first, the molecular weight was more substantial. Therefore, the change in the molecular weight would be more obvious, which could be correlated with the degradation of the polymer. With hydrolysis, the molecular weight of the following hydrolyzed product of the polymer would be smaller, so the change of molecular weight would become less significant, with a continuation of the degradation time [57]. The molecular weight of the scaffolds in the first four weeks of the degradation was reduced, rapidly. From the fifth-week, the decrease of molecular weight had slowed down, due to the decrease in the cleavage sites of the polymer with a smaller molecular weight. Therefore, the amplitude of the molecular weight decreased with an increase in the incubation period. Moreover, as the degradation proceeded, the range of molecular weight distribution would widen, gradually, due to the difference of the degradation speed, which can be observed in Figure 6C,D. After ten weeks of degradation, the molecular weight (Mw) of the polymer in enzymolysis and hydrolysis degradation study, was 8734 kDa and 7867 kDa respectively. On the other hand, due to ester bond cleavage, exposed hydrophilic groups (carboxyl and hydroxyl groups) had formed on the surface of the polymer, which might have enhanced the surface hydrophilicity of the scaffold. This behavior subsequently favored the cell adhesion and growth appropriately. With the degradation of the PLGA scaffolds in the medium, the wetting behavior might increase gradually, due to the enhancement of the hydrophilicity of the PLGA scaffolds, during the degradation process [58]. The thermodynamic properties of materials reflect the changing trend of the composition as well as properties during the process of degradation. It can be seen from Figure 6E,F, that the thermal decomposition temperature of the scaffold, in the enzymatic and hydrolytic studies, gradually decreased from 359.42 to 302.28 and 360.43 to 304.56 °C, respectively. This happened due to the decrease in the regularity of the polymer molecular chain leading to the decrease of the molecular weight, which subsequently weakened the intermolecular forces, and the decrease of thermal decomposition temperature.

### 3.3. Bioefficacy of the PLGA Scaffolds

#### 3.3.1. Cytotoxicity Test

**Figure 7:**
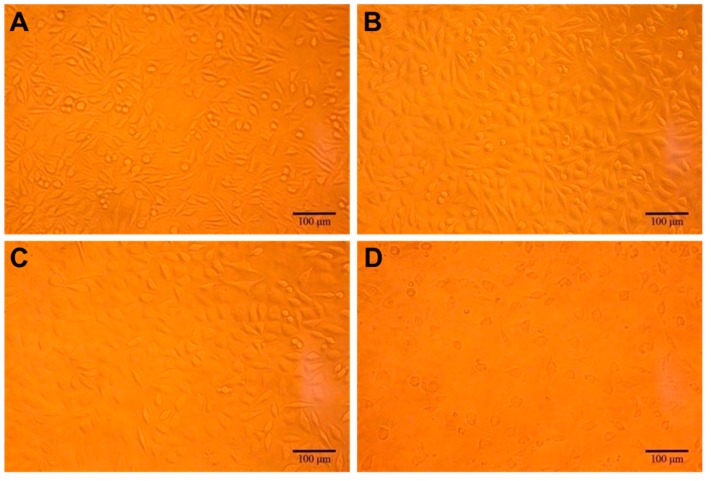
An optical micrograph of L929 cells cultured for 72 h, at different concentrations of 3D printed PLGA scaffold extract. (**A**) Negative control, (**B**) 50 mg/mL, and (**C**) 100 mg/mL of the PLGA scaffolds as the treatment group, and (**D**) Positive control (Phenol).

**Figure 8:**
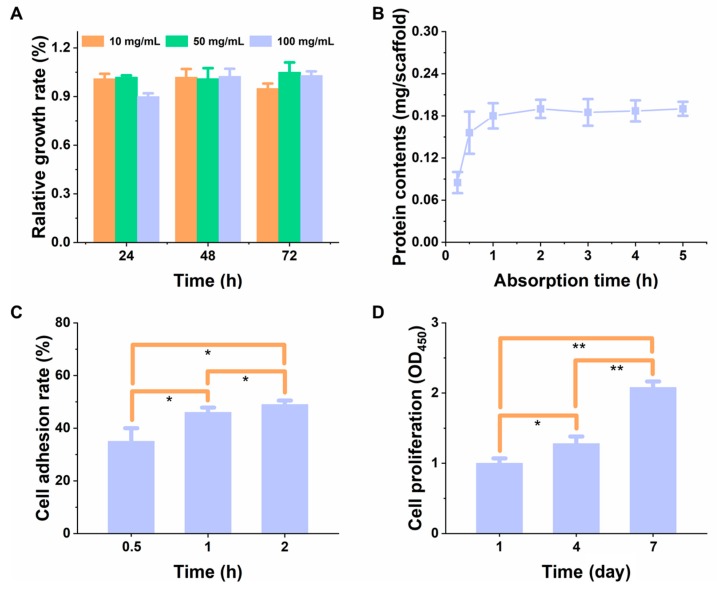
(**A**) The relative growth rate of L929 cells on the PLGA scaffold. The concentrations of the scaffold leaching solution were 10, 50, and 100 mg/mL, respectively. (**B**) Protein adsorption efficacy onto the scaffold at different time intervals. (**C**) Cell adherence rate and (**D**) proliferation rate on the scaffold, at different time intervals, (*n* = 5). * represents *p <* 0.05, ** represents *p <* 0.01.

Cytotoxicity refers to the cytotoxic events caused by the substances released from the material, when co-cultured with the cells, which could be evaluated through different methods [19]. At first, the cytotoxicity test was assessed, qualitatively, by capturing the micrographs of L929 cells (normal fibroblasts), using an optical microscope, as this cell line is the most preferred typical model for measuring compatibility issues of the cells [35]. In the control group treatment, it showed spindle-shaped or irregular triangle, at a high density, with a clear boundary and normal pseudopodia after being attached to the wall (Figure 7). The experimental group of the scaffold extract solution, showed that the cell morphology and density of the cells were consistent with the negative control group, and there was no obvious difference in the density of cells. It showed that the cells had no morphological and quantitative changes with the increase of the concentration of the extract, revealing that the scaffold was non-toxic. Contrarily, in the positive control treatment group, the cell density decreased in terms of the circular cell morphology, and a small amount of cell debris, indicating that the toxic effects of phenol on the cells affect the normal growth of the cells. Further, the quantitative evaluation of the cytotoxic effects of the scaffolds, on L929 cells, was determined. In Figure 8A, the proliferation rate of L929 cells was compared by co-culturing the cells with different concentrations of the scaffold extract (10, 50, and 100 mg/mL), in normal culture conditions. It was evident from the results that the cell proliferation rate at different concentrations of the scaffold extract was more than 91% after 24, 48, and 72 h of incubation, indicating that there was no obvious inhibitory effect of the scaffold extract on the cells. The excellent and promising cell growth explicitly shows that these scaffolds are highly suitable for tissue regeneration materials. This data is in accordance with the results of Kankala and colleagues, where MC3T3-E1 and BMSC cells, at different exposure times of 24, 48, and 72 h, showed no cytotoxicity.

#### 3.3.2. The Relative Proliferation Rate of Cells and Adsorption Kinetics of Protein

Prior to the adhesion and the growth of cells on the PLGA scaffolds, the protein adsorption ability on the scaffold surface was measured, which is helpful to understand the protein adsorption kinetics and to correlate with the cell adhesion behavior of the biocompatible TE scaffolds [59]. Figure 8B illustrates the adsorption kinetic curve of the BSA protein on the scaffold. It was evident from the data, that the amount of protein adsorbed on the scaffold increased with time and reached a dynamic equilibrium in about 1 h, and was in a dynamic equilibrium state, within the subsequent 4 h of incubation (0.19 ± 0.01 mg/scaffold), indicating that the scaffold possessed excellent protein adsorption ability and was highly suitable for cell adhesion and growth [60]. Furthermore, the ECM formation and subsequent contribution of cells towards it, might have influenced the cell adhesion and proliferation. The formation of the ECM was determined by measuring the protein secretion of the cells on the scaffold. The results showed that after the co-culturing of the cells, with the scaffolds, the protein secretion was 1.02 *±* 0.04 mg/scaffold, demonstrating that cells could secrete proteins on scaffolds and facilitate the ECM formation, significantly.

The cell counting method was used to determine the rate of cell adhesion on the scaffold surface, at different exposure times of 0.5, 1, and 2 h. The experimental results showed that the adherence rate of cells on the scaffold had gradually increased with time (Figure 8C). Then, the CCK-8 assay was used to determine the proliferation of cells quantitatively (Figure 8D). It was evident that the viability of cells increased gradually, within a week of culturing process of the cells and the scaffolds, indicating that the number of cells was always in a state of growth and could generally proliferate on the scaffold. Therefore, the investigation of cell adhesion and proliferation showed that the cells could adhere and proliferate on the scaffold, and the cell concentration increased with an increase in time, which could be attributed to the excellent compatibility of the scaffolds [61].

#### 3.3.3. Cell Distribution Study

**Figure 9:**
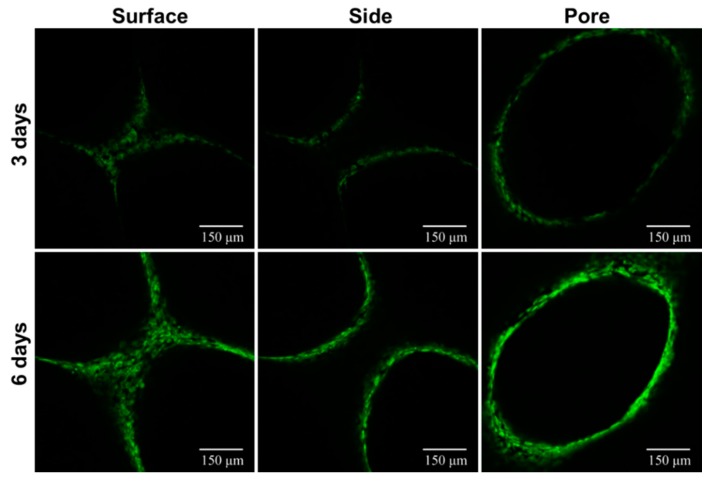
CLSM images of MC3T3-E1 cells-scaffold composites, for determining the distribution of the cells on the scaffolds.

To understand the growth, as well as the distribution of cells on the scaffold, more intuitively, an acridine orange solution was used to stain the scaffold-cell complex, after 3 and 6 days of incubation, and then the scaffolds were observed under the CLSM. The images showed that the cells grew normally on the scaffold and distributed more evenly (Figure 9). With the increase of the culture time, the number of cells increased gradually, and the activity was high, which could be attributed to the biocompatibility of the scaffolds, which played a crucial role in promoting the healthy growth of cells.

## 4. Conclusions

In summary, we fabricated PLGA scaffolds, using FDM-based 3D-printing approach, and critically evaluated various physicochemical attributes, which claimed its suitability for bone TE. In this study, the critical problems addressed were the cumbersome preparation, poor reproducibility, low preparation speed, and the residual solvents in the preparation of bone substitute materials. Moreover, the scaffolds have exhibited excellent degradation ability, in enzymatic and hydrolytic-based buffers, as well as mechanical properties suitable for cancellous bone formation. Furthermore, these scaffolds have exhibited excellent biocompatibility, which has promoted the proliferation, growth, and excellent distribution of preosteoblasts in the scaffolds. This critical evaluation of the various physicochemical attributes would undoubtedly provide an enormous scope in generating 3D scaffolds by using the 3D-printing approach.

## Figures and Tables

**Table 1:** Various printing parameters of the PLGA scaffolds.

Nozzle Inner Diameter (mm)	Temperature (°C)	Pressure (MPa)	Velocity (mm/s)	Layer Thickness (mm)	Filling Distance (mm)
0.2	215 *±* 2	0.15–0.3	8–11	0.17	0.8–1.1
0.3	205 *±* 2	0.1–0.25	8–11	0.25	0.8–1.2
0.4	197 *±* 2	0.08–0.2	10–12	0.33	0.9–1.2

**Table 2:** Pore size and a wire diameter of the scaffolds, printed at three different angles.

Structures	0°/90°	0°/60°/120°	0°/45°/90°/135°
Pore size (μm)	575–742	584–775	443–761
Wire diameter (μm)	221–342	205–268	205–322
